# Mesenchymal stem cells genetically modified by lentivirus-mediated interleukin-12 inhibit malignant ascites in mice

**DOI:** 10.3892/etm.2014.1918

**Published:** 2014-08-19

**Authors:** JIMING HAN, JUMEI ZHAO, JIANRONG XU, YANJUN WEN

**Affiliations:** 1 Medical College of Yan’an University, Yan’an, Shaanxi 716000, P.R. China; 2 Southwest University of Science and Technology, Mianyang, Sichuan 621010, P.R. China; 3 State Key Laboratory of Biotherapy, West China Hospital, Sichuan University, Chengdu, Sichuan 610041, P.R. China

**Keywords:** interleukin-12, malignant ascites, mesenchymal stem cells, immunotherapy

## Abstract

The objective of the present study was to investigate the effects of mesenchymal stem cells (MSCs) genetically modified by lentivirus-mediated mouse interleukin-12 (Lenti-mIL-12) in treating malignant ascites in mice. The *in vitro* chemotactic effect of Lenti-mIL-12-MSC culture supernatant on dendritic cells was investigated using a chemotaxis chamber. Liver cancer H22 and MethA ascites models were constructed. Mice were divided evenly into four groups: Normal saline, MSC, Null and Lenti-mIL-12-MSC. The survival rate, ascites volume and red blood cell number were measured for these groups. The toxicity and side effects of Lenti-mIL-12-MSCs were investigated using visual and microscopy inspections. The results indicated that mIL-12 had a strong chemotactic effect on dendritic cells. mIL-12 was highly expressed in ascites of Lenti-mIL-12-MSC-treated mice. Lenti-mIL-12-MSCs reduced the volume of ascites and the number of red blood cells in ascites and thus increased the survival rate and prolonged the survival duration of the mice. Furthermore, Lenti-mIL-12-MSCs showed no toxicity and side effects on the mice with malignant ascites. In conclusion, the results demonstrated that Lenti-mIL-12-MSCs inhibited the growth of ascites and promoted the survival of tumor-bearing mice, suggesting that Lenti-mIL-12-MSCs exerts a therapeutic effect on malignant ascites by stimulating the immune responses of the mice.

## Introduction

Malignant ascites, a common complication of abdominal malignancies, is one of the main causes of mortality in patients with cancer. The treatment of malignant ascites is important for improving the prognosis of abdominal malignancies. There are numerous methods for the treatment of malignant ascites; at present, the frequently used methods include intraperitoneal chemotherapy, intraperitoneal injection of radioisotopes or biological response modifiers, systemic chemotherapy and radiotherapy (1–3). However, specifically effective therapies remain lacking, and existing problems include short efficacy and drug resistance, as well as questions on how to maintain high levels of abdominal local drug concentration. With the development of modern medical technology in gene therapy and immunotherapy, immunogene therapy has become one of the most promising anti-tumor therapeutic strategies (4,5).

Mononuclear cells, important members of the body immune response, have the potential to differentiate into macrophages and dendritic cells. Interleukin-12 (IL-12) is an interleukin that is naturally produced by dendritic cells and macrophages in response to anti-tumor immune stimulation. IL-12 exhibits certain anti-tumor effects by inhibiting tumor growth and prolonging the survival time of tumor-bearing animals (6,7). However, in practical application, numerous problems remain with IL-12 treatment, including large doses, repeated injections and a number of side-effects (8). It is important to select an improved therapy carrier to limit IL-12 entering the tumor tissue and to maintain its therapeutic dose for tumor therapy. Among the potential carriers, mesenchymal stem cells play an important role in the targeted release of therapeutic gene expression products into the tumor tissue to inhibit tumor growth and metastasis. In the present study, the mouse interleukin (mIL)-12 gene was introduced into mesenchymal stem cells (MSCs) via the mediation of lentivirus, and highly efficient and stable MSCs were obtained through screening. Using MSCs as the targeted vehicle of gene therapy, the anti-ascites effect of lentivirus-mediated mIL-12 MSCs (Lenti-mIL-12-MSCs) was explored.

## Materials and methods

### Cells and animals

The MethA fibrosarcoma and the transplanted H22 hepatoma cell lines were purchased from the American Type Culture Collection (Manassas, VA, USA). The Lenti-mIL-12-MSC stable cell line was constructed and preserved by the State Key Laboratory of Biotherapy at the West China Hospital (Sichuan University, Chengdu, China). Female BALB/c mice (6–8 weeks old) were purchased from the West China Experimental Animal Center of Sichuan University (Chengdu, China). All animal experiments were conducted according to the ethical guidelines of Sichuan University. The present study was approved by the Institutional Review Board and Ethics committee of the Medical College of Yan’an University (Yan’an, China).

### Isolation and chemotaxis of mouse bone marrow MSCs

Bone marrow cells were washed out by RPMI-1640 medium from the femur of BALB/c mice isolated in a sterile environment. Subsequent to centrifugation and the disposal of supernatants, sterile Tris-NH_4_Cl was added prior to another centrifugation that lysed the red blood cells. Tris-NH_4_Cl was then rinsed off and the cells were pelleted by centrifugation. The cells were subsequently suspended in RPMI-1640 medium containing 10 ng/ml granulocyte-macrophage colony-stimulating factor, IL-4 and 10% fetal calf serum. The cell suspension was transferred into six-well culture plates for incubation at 37°C in 5% CO_2_. After 48 h, the medium was changed, during which time the suspended cells were discarded. The cells were collected after five days of culture.

A chemotaxis chamber (96-well format, Neuro Probe, Inc., Gaithersburg, MD, USA) was placed into 0.5 mol/l acetic acid for 2 min and then phosphate-buffered saline (PBS) was used to wash off the acetic acid. Subsequent to soaking with 0.1% gelatin for 2 h, the chamber was naturally dried. The cells were divided into MSC (untreated MSCs), Null (MSCs screened following the transfection with empty virus) and Lenti-mIL-12-MSC (MSCs screened following the transfection with Lenti-mIL-12) groups. The supernatant of serum-free cell culture was centrifuged and the volume was reduced to one-fifth of the original volume. The concentrated supernatant from each group (50 μl) was added into the lower layer of the wells in triplicate. Dendritic cells (30 μl, ~1×10^6^/ml) were subsequently added above the polyvinylpyrrolidone (PVP) membrane and incubated at 37°C for 2 h. Following incubation, the upper layer of the PVP membrane was washed with PBS and fixed by ethanol. The number of cells that migrated to the lower layer was counted under high magnification following Wright-Giemsa’s staining. Six fields of vision were used for the cell count for each well.

### Enzyme-linked immunosorbent assay (ELISA)

The level of IL-12 was measured using the ELISA kit (BD Biosciences, Bedford, MA, USA). The procedure was carried out according to the manufacturer’s instructions.

### Observation of survival rate and duration for tumor-bearing mice

Inbred female BALB/c mice (6–8 weeks old) were used as hosts for transplanted H22 hepatoma and MethA fibrosarcoma cells, and were fed in standardized sterile isolation cages. Ascites models were constructed as follows (5): MethA cells (1×10^6^ cells/mouse) were inoculated into the peritoneal cavity of 40 mice, which were randomly divided into four groups of 10 mice on the second day. The four groups were peritoneally injected on days 2 and 7 after inoculation with: i) 200 μl saline [normal saline (NS) group]; ii) 200 μl uninfected MSCs (total cell number, 2×10^6^; MSC group); iii) 200 μl infected Lenti-Null-MSCs (total cell number, 2×10^6^; Null group); iv) 200 μl infected Lenti-mIL-12-MSCs (total cell number, 2×10^6^; Lenti-mIL-12-MSC group). The status of ascites development was measured every two days and the survival rate (the percentage of surviving mice in each group) was observed until day 40 (only the Lenti-mIL-12-MSC group had surviving mice by this time). The construction, grouping, treatment and inoculation for the H22 model were performed in an identical manner.

### Determination of ascites volume and red blood cell number for tumor-bearing mice

The grouping and treatment were performed as described above. On day 12 after the inoculation, the mice were sacrificed and the ascites was collected to measure the volume, followed by centrifugation. The supernatants and cells were collected, and red blood cell number was counted under the microscope.

### Observation of toxicity and side effects

During the treatment, the general conditions of the activities, feeding, fur and body weight of the ascites-bearing mice were observed. Following treatment, the mice were sacrificed by cervical dislocation. The heart, liver, spleen, kidney, pancreas, small intestines, brain, lungs and bone marrow of the mice were visually inspected and prepared into biopsies for observation under optical microscopes following hematoxylin and eosin staining.

### Statistical analysis

All data are expressed as the mean ± standard deviation. Statistical analyses were performed by Student’s t-test using SPSS 17.0 for Windows (StatSoft Inc., Tulsa, OK, USA). The Kaplan-Meier curves for mice survival rates were analyzed by log-rank test. The test level was α=0.05, and P<0.05 was considered to indicate a statistically significant difference.

## Results

### mIL-12 exerts a strong chemotactic effect on dendritic cells

**Figure 1:**
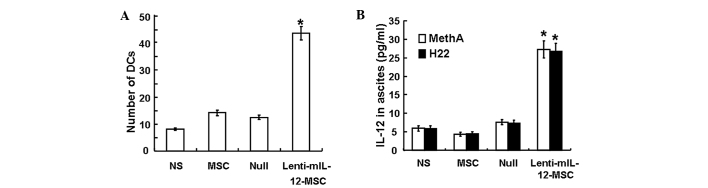
(A) *In vitro* chemotactic effect of Lenti-mIL-12-MSC culture supernatant on DCs. The histogram shows the number of DCs that passed through the membrane from the upper layer of the chemotaxis chamber to the lower layer. Data are presented as the mean ± SD (n=3). ^*^Significant difference from the NS, MSC and Null controls in the same model (P<0.01). (B) Concentrations of mIL-12 in ascites in the MethA and H22 models. Data are presented as the mean ± SD (n=10). ^*^Significant difference from the NS, MSC and Null controls in the same model (P<0.01). NS, MSC, Null and Lenti-mIL-12-MSC groups were peritoneally injected with 200 μl saline, 200 μl uninfected MSCs (total cell number 2×10^6^), 200 μl infected Lenti-Null-MSCs (total cell number 2×10^6^) or 200 μl infected Lenti-mIL-12-MSCs (total cell number 2×10^6^), respectively. Lenti-mIL-12, lentivirus-mediated mouse interleukin-12; MSC, mesenchymal stem cell; NS, normal saline; DC, dendritic cell; SD, standard deviation.

To assess the chemotactic effect of mIL-12 on dendritic cells, the number of dendritic cells that migrated through the PVP membrane in the chemotaxis chamber was counted under the microscope following Wright-Giemsa’s staining. The average number of cells that passed through the PVP membrane and were counted under each high magnification field was 8±0.4 for the NS group, 14±0.9 for the MSC group, 12±0.8 for the Null group and 43±2.4 for the Lenti-mIL-12-MSC group (Fig. 1A). Statistical analysis indicated that the dendritic cell chemotactic effect of supernatants from the Lenti-mIL-12-MSC group cell culture was stronger than that of supernatants from other groups (P<0.01). This result suggested that mIL-12 exhibited a strong chemotactic effect on dendritic cells.

### mIL-12 is highly expressed in ascites of Lenti-mIL-12-MSC-treated mice

To assess the expression of IL-12 in mouse ascites, IL-12 concentration was measured using ELISA. In both the MethA and H22 tumor models, the concentration of IL-12 in the ascites of the Lenti-mIL-12-MSC group (30 pg/ml) was significantly higher than that of the control groups (<7.5 pg/ml) (P<0.05), whereas no significant differences were observed among the control groups (P>0.05) (Fig. 1B). The results revealed that Lenti-mIL-12-MSCs promoted the expression of IL-12 in ascites.

### Lenti-mIL-12-MSCs reduce the volume of ascites and the number of red blood cells

**Figure 2:**
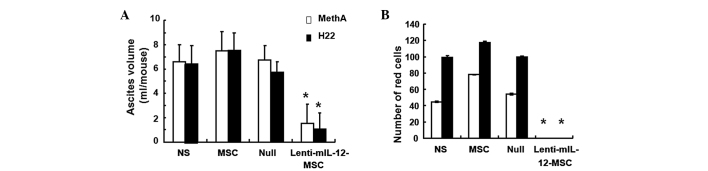
Effect of Lenti-mIL-12-MSC on the (A) volume of ascites and (B) number of red blood cells in the MethA and H22 models. Data are presented as the mean ± standard deviation (n=10). ^*^Significant difference from the NS, MSC and Null controls in the same model (P<0.01). NS, MSC, Null and Lenti-mIL-12-MSC groups were peritoneally injected with 200 μl saline, 200 μl uninfected MSCs (total cell number 2×10^6^), 200 μl infected Lenti-Null-MSCs (total cell number 2×10^6^) or 200 μl infected Lenti-mIL-12-MSCs (total cell number 2×10^6^), respectively. Lenti-mIL-12, lentivirus-mediated mouse interleukin-12; MSC, mesenchymal stem cell; NS, normal saline.

To investigate how Lenti-mIL-12-MSCs affect ascites volume and red blood cell number, ascites was collected for volume measurement and red blood cells were counted. The observations indicated that the control groups of mice not treated with Lenti-mIL-12-MSCs showed reduced food and water intake, dullness, inactivity, poor reactivity and rapidly growing ascites in the early stage, and had extreme abdominal distension, emaciation, cachexia and a large amount of peritoneal viscous and bloody ascites in the advanced stage. By contrast, the Lenti-mIL-12-MSC group of mice exhibited good responsiveness, no abdominal distension, low viscosity and blood-free ascites. In addition, the data showed that the volume of ascites and the red blood cell count in the Lenti-mIL-12-MSC group were significantly lower than those in the control groups (P<0.01) (Fig. 2). These results demonstrated that Lenti-mIL-12-MSCs inhibited the formation of ascites in the MethA and H22 models, with significantly reduced severity of malignancy.

### Lenti-mIL-12-MSCs increase the survival rate and prolong the survival duration of the mice

**Figure 3:**
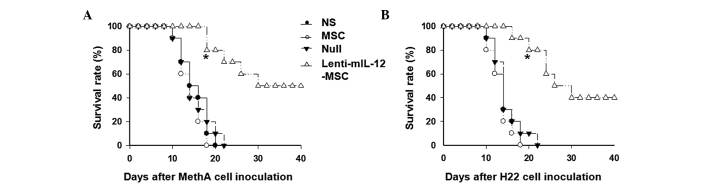
Effect of Lenti-mIL-12-MSC on the survival rate of mice in the (A) MethA and (B) H22 models. Data are presented as the mean ± standard deviation (n=10). ^*^Significant difference from the NS, MSC and Null controls in the same model (P<0.01). NS, MSC, Null and Lenti-mIL-12-MSC groups were peritoneally injected with 200 μl saline, 200 μl uninfected MSCs (total cell number 2×10^6^), 200 μl infected Lenti-Null-MSCs (total cell number 2×10^6^) or 200 μl infected Lenti-mIL-12-MSCs (total cell number 2×10^6^), respectively. Lenti-mIL-12, lentivirus-mediated mouse interleukin-12; MSC, mesenchymal stem cell; NS, normal saline.

To understand how Lenti-mIL-12-MSCs affect the survival of mice, the survival rate was observed and calculated until day 40 after the inoculation. During the experiment, the body weight and abdominal circumference of the mice in the control groups rapidly increased, and these mice started exhibiting high mortality rates 10 days after the inoculation. However, the mice in the Lenti-mIL-12-MSC group survived and lived healthily for a longer time, with slow increases in body weight and abdominal circumference. The survival time of the mice in the Lenti-mIL-12-MSC group was prolonged compared with that in the control groups. When all the mice in MSC group had died, 80% of the mice in the Lenti-mIL-12-MSC group remained alive (P<0.01) (Fig. 3). These results suggested that Lenti-mIL-12-MSCs increased the survival rate and prolonged the survival duration of the mice.

### Lenti-mIL-12-MSCs have no toxicity and side effects on tumor-bearing mice

To assess the toxicity and side effects of Lenti-mIL-12-MSC injection, observations were made on the general condition and hematoxylin and eosin-stained biopsies of internal organs of the mice in each group. These observations revealed no physiological and pathological abnormalities (data not shown), suggesting that Lenti-mIL-12-MSCs exhibited neither toxicity nor side effects on tumor-bearing mice.

## Discussion

IL-12, a T-cell-stimulating factor, promotes the differentiation and proliferation of CD4^+^, T and natural killer cells, as well as interferon-γ production in these cells. IL-12 also increases the activity of lymphokine-activated killer cells (9) and induces anti-angiogenic activities (10). These properties indicate that IL-12 has potential as an immunomodulatory factor for the treatment of malignant tumors. In various tumor models, IL-12 has already shown promising anti-tumor effects that prevent tumor formation, growth and metastasis (11–13).

A key point in gene therapy is how to introduce the target gene and highly express the target protein in the targeted cells. Gene transduction can be achieved by physical, chemical and biological techniques, including direct injection of plasmid DNA, gene gun technology, electroporation and transfection by liposomes or viruses. There has been a recent gradual increase in the use of cell vehicles in gene therapies of tumors. Bone marrow MSCs (BMMSCs) are a type of adult stem cell that mainly exists in adult bone marrow. These cells have very low immunogenicity and are not rejected in allografts or heterografts. In addition, BMMSCs exhibit pluripotency and a strong proliferation ability, and can directionally migrate to tumor lesions (7,14,15).

In the present study, the mIL-12 gene was introduced into MSCs via the mediation of lentivirus, and highly efficient and stable MSCs were obtained through screening (16). Using MSCs as the targeted vehicle of gene therapy, the anti-ascites effect of Lenti-mIL-12-MSCs was explored. *In vitro* chemotaxis experiments showed that the culture supernatant of MSCs expressing mIL-12 had a strong chemotactic effect on dendritic cells. Compared with mice in the other groups, malignant ascites-bearing mice treated with Lenti-mIL-12-MSCs exhibited a smaller ascites volume, and a lower red blood cell number and viscosity, as well as a significantly prolonged survival duration and rate, suggesting that this system could inhibit ascites formation in the mice. These results indicated that the chemotactic and maturity-inducing effect of mIL-12 on dendritic cells effectively stimulated anti-tumor immunity in the body. Therefore, the inhibition of ascites by injected MSCs expressing mIL-12 was mediated by immune responses. In addition, the use of lentivirus as a vehicle prolonged the expression duration of IL-12 in the abdomen. This may provide novel avenues for the treatment of malignant ascites.

## References

[1] Scheithauer W, Kornek GV, Raderer M (2003). Randomized multicenter phase II trial of two different schedules of capecitabine plus oxaliplatin as first-line treatment in advanced colorectal cancer. J Clin Oncol.

[2] Santini D, Massacesi C, D’Angelillo RM (2004). Raltitrexed plus weekly oxaliplatin as first-line chemotherapy in metastatic colorectal cancer: a multicenter non-randomized phase ii study. Med Oncol.

[3] Sartori S, Nielsen I, Tassinari D, Trevisani L, Abbasciano V, Malacarne P (2001). Evaluation of a standardized protocol of intracavitary recombinant interferon alpha-2b in the palliative treatment of malignant peritoneal effusions. A prospective pilot study. Oncology.

[4] Walther W, Schlag PM (2013). Current status of gene therapy for cancer. Curr Opin Oncol.

[5] Yoshiji H, Kuriyama S, Hicklin DJ (2001). The vascular endothelial growth factor receptor KDR/Flk-1 is a major regulator of malignant ascites formation in the mouse hepatocellular carcinoma model. Hepatology.

[6] Cheema TA, Fecci PE, Ning J, Rabkin SD (2014). Immunovirotherapy for the treatment of glioblastoma. Oncoimmunology.

[7] Marchi LH, Paschoalin T, Travassos LR, Rodrigues EG (2011). Gene therapy with interleukin-10 receptor and interleukin-12 induces aprotective interferon-γ-dependent response against B16F10-Nex2 melanoma. Cancer Gene Ther.

[8] Quetglas JI, Dubrot J, Bezunartea J (2012). Immunotherapeutic synergy between anti-CD137 mAb and intratumoral administration of a cytopathic Semliki Forest virus encoding IL-12. Mol Ther.

[9] Dickerson EB, Akhtar N, Steinberg H (2004). Enhancement of the antiangiogenic activity of interleukin-12 by peptide targeted delivery of the cytokine to alphavbeta3 integrin. Mol Cancer Res.

[10] Wigginton JM, Gruys E, Geiselhart L (2001). IFN-gamma and Fas/FasL are required for the antitumor and antiangiogenic effects of IL-12/pulse IL-2 therapy. J Clin Invest.

[11] Zhang L, Kerkar SP, Yu Z (2011). Improving adoptive T cell therapy by targeting and controlling IL-12 expression to the tumor environment. Mol Ther.

[12] Kerkar SP, Muranski P, Kaiser A (2010). Tumor-specific CD8^+^ T cells expressing interleukin-12 eradicate established cancers in lymphodepleted hosts. Cancer Res.

[13] Parker JN, Meleth S, Hughes KB, Gillespie GY, Whitley RJ, Markert JM (2005). Enhanced inhibition of syngeneic murine tumors by combinatorial therapy with genetically engineered HSV-1 expressing CCL2 and IL-12. Cancer Gene Ther.

[14] Dai LJ, Moniri MR, Zeng ZR (2011). Potential implications of mesenchymal stem cells in cancer therapy. Cancer Lett.

[15] Bexell D, Svensson A, Bengzon J (2013). Stem cell-based therapy for malignant glioma. Cancer Treat Rev.

[16] Hu YL, Fu YH, Tabata Y, Gao JQ (2010). Mesenchymal stem cells: a promising targeted-delivery vehicle in cancer gene therapy. J Control Release.

